# Impact of viral coinfection and macrolide-resistant mycoplasma infection in children with refractory *Mycoplasma pneumoniae* pneumonia

**DOI:** 10.1186/s12879-020-05356-1

**Published:** 2020-08-26

**Authors:** Yajuan Zhou, Jing Wang, Wenjuan Chen, Nan Shen, Yue Tao, Ruike Zhao, Lijuan Luo, Biru Li, Qing Cao

**Affiliations:** 1 Department of Infectious Diseases, Shanghai Children’s Medical Center, Shanghai Jiaotong University School of Medicine, Shanghai, China; 2 The Laboratory of Pediatric Infectious Diseases, Pediatric Translational Medicine Institute, Shanghai Children’s Medical Center, Shanghai Jiaotong University School of Medicine, Shanghai, China; 3 Department of Critical Care Medicine, Shanghai Children’s Medical Center, Shanghai Jiaotong University School of Medicine, Shanghai, China

**Keywords:** *Mycoplasma pneumoniae*, Coinfection, FilmArray respiratory panel, Children

## Abstract

**Background:**

Cases of refractory *Mycoplasma pneumoniae* pneumonia have been increasing recently; however, whether viral coinfection or macrolide-resistant *M.* infection contribute to the development of refractory *M. pneumoniae* pneumonia remains unclear. This study aimed to investigate the impacts of viral coinfection and macrolide-resistant *M. pneumoniae* infection on *M. pneumoniae* pneumonia in hospitalized children and build a model to predict a severe disease course.

**Methods:**

Nasopharyngeal swabs or sputum specimens were collected from patients with community-acquired pneumonia meeting our protocol who were admitted to Shanghai Children’s Medical Center from December 1, 2016, to May 31, 2019. The specimens were tested with the FilmArray Respiratory Panel, a multiplex polymerase chain reaction assay that detects 16 viruses, *Bordetella pertussis*, *M. pneumoniae*, and *Chlamydophila pneumoniae*. Univariate and multivariate logistic regression models were used to identify the risk factors for adenovirus coinfection and macrolide-resistant mycoplasma infection.

**Results:**

Among the 107 *M. pneumoniae* pneumonia patients, the coinfection rate was 56.07%, and 60 (60/107, 56.07%) patients were infected by drug-resistant *M. pneumoniae*. Adenovirus was the most prevalent coinfecting organism, accounting for 22.43% (24/107). The classification tree confirmed that viral coinfection was more common in patients younger than 3 years old. Adenovirus coinfection and drug-resistant *M. pneumoniae* infection occurred more commonly in patients with refractory *M. pneumoniae* pneumonia (*P* = 0.019; *P* = 0.001). A prediction model including wheezing, lung consolidation and extrapulmonary complications was used to predict adenovirus coinfection. The area under the receiver operating characteristic curve of the prediction model was 0.795 (95% CI 0.679–0.893, *P* < 0.001). A prolonged fever duration after the application of macrolides for 48 h was found more commonly in patients infected by drug-resistant *M. pneumoniae* (*P* = 0.002). A fever duration longer than 7 days was an independent risk factor for drug-resistant *Mycoplasma* infection (OR = 3.500, 95% CI = 1.310–9.353, *P* = 0.012).

**Conclusions:**

The occurrence of refractory *M. pneumoniae* pneumonia is associated with adenovirus coinfection and infection by drug-resistant *M. pneumoniae*. A prediction model combining wheezing, extrapulmonary complications and lung consolidation can be used to predict adenovirus coinfection in children with *M. pneumoniae* pneumonia. A prolonged fever duration indicates drug-resistant *M. pneumoniae* infection, and a reasonable change in antibiotics is necessary.

## Background

*Mycoplasma pneumoniae* (MP) is a common pathogen that causes community-acquired pneumonia in children [1]. MP infection is considered a self-limited disease. However, it has been verified that MP infection can progress into severe disease in some cases [2]. Increasing numbers of refractory or severe *Mycoplasma pneumoniae* pneumonia (MPP) cases have been reported worldwide, especially in Asia [2, 3]. Previous studies have shown that refractory *Mycoplasma pneumoniae* pneumonia (RMPP) is associated with prolonged fever, high levels of C-reactive protein (CRP), airway hypersecretion and consolidation on chest imaging [4, 5]. It has been confirmed that the excessive immune response of the host plays an important role in the development of RMPP [5, 6]. However, whether different features of pathogens contribute to the development of RMPP remains unclear. Previous research confirmed that coinfection with viruses and bacteria led to more severe disease in children with RMPP [7]. In addition, macrolide-resistant MP infection may also play an important role in the occurrence and development of RMPP [8].

The purpose of this study was to investigate the impacts of viral coinfection and macrolide-resistant MP infection on hospitalized MPP patients, to identify the risk factors for these possible impacts, and then to build a model to predict a severe disease course.

## Methods

### Subjects and specimens

The study was performed from December 1, 2016, to May 31, 2019, at Shanghai Children’s Medical Center (SCMC), a tertiary care hospital in Shanghai. Patients was enrolled according to the inclusion criteria: (1) patients with symptoms such as: fever, cough, wheezing; (2) physical examination revealed rales in the lungs; (3) imaging examination confirmed inflammation in the lungs. Patients with MPP were diagnosed according to the clinical practice guidelines for the management of community-acquired pneumonia in infants and children and the British Thoracic Society guidelines for the management of community-acquired pneumonia in children updated in 2011 [9, 10]. RMPP was defined as: (1) a sustained fever for 7 days or more and; (2) increasingly severe cough and infiltrates on chest radiographs despite the administration of appropriate macrolide antibiotics. MP was detected by FilmArray Respiratory Panel (FilmArray RP), a multiplex polymerase chain reaction (PCR) assay that detects 16 viruses, MP, *Bordetella pertussis (B. pertussis),* and *Chlamydophila pneumonia (C. pneumoniae)*. Children with congenital or secondary immunodeficiency were excluded.

Nasopharyngeal swabs (NPSs) or sputum specimens were collected from patients once they were admitted to our hospital and MP-IgM was tested at the same time. The medical records of each patient, including demographic data, clinical features, laboratory tests and radiological results, were obtained. The study was approved by the Institutional Review Board of Shanghai Children’s Medical Center (SCMCIRB-K2017044), and written informed consent was obtained from the parents of each patient.

### FilmArray RP v 1.7 testing

The FilmArray Respiratory Panel v1.7 detects 19 pathogens: adenovirus (ADV); influenza A viruses H1, 2009H1, H3 (FluA-H1, FluA-2009H1, FluA-H3) and FluB; parainfluenza virus types 1 to 4 (Para 1–4); coronaviruses 229E, HKU1, OC43, and NL63 (Cov-HKU1, NL63, 229E, OC43); human metapneumovirus (hMPV); respiratory syncytial virus (RSV); human rhinovirus/enterovirus (Rhino/Entero); *C. pneumoniae*); *MP*; and *B. pertussis*. The FilmArray Respiratory Panel assay was performed according to the manufacturer’s instructions. The principle of the assay has been previously described [11–13].

### Detection of MP drug-resistant genes

Nasopharyngeal aspirates were collected at admission and assayed for MP DNA copy number using the QIAamp DNA MINI kit (QIAGEN, Germany). The sequence of the drug resistance locus was retrieved from GenBank, and primers were designed to amplify the drug resistance locus. The amplified product length was approximately 150 bp. The primer sequences were as follows: MP 1F: AACTATAACGGTCCTAAGGTAGCG, MP 2R: GCTCCTACCTATTCTCTACATGAT. Each reaction contained 0.125 l of EX tag HS enzyme, 2.5 l of dNTP, 2.5 l of 10*EX tag buffer, 0.5 l of Primer F, 0.5 l of Primer R, 2 l of template DNA and ddH2O to achieve a final volume of 25 l. The cycling conditions were as follows: 94 °C for 5 min, followed by 35 cycles of 94 °C for 20 s, 55 °C for 30 s, and 72 °C for 30 s with a final extension step of 72 °C for 5 min. The DNA product was then sequenced by an external vendor (BGI, Shanghai, China), and the sequencing results were submitted to NCBI blast for analysis. The nucleotide at position 2063 was A for wild type but G for the mutant. The 23S rRNA domain V was amplified by nested PCR using a Veriti® 96-Well Thermal Cycler (Singapore). Finally, the DNA sequences were obtained (ABI, America).

### Serological testing

To detect MP antibodies, commercially available IgM indirect immunofluorescence assays (IFA) were performed (Pneumoslide IgM, Vircell, S.L., Spain). Serological diagnosis was defined by the appearance of green fluorescence around the cell.

### Statistical analysis

A *P* value< 0.05 was considered statistically significant. Categorical variables are expressed as frequencies and percentages. The chi-square test was used to compare groups. Continuous variables are expressed as the means and standard deviations, and comparisons were made using Student’s t-tests. Variables that had a *P* value< 0.05 in the univariate logistic analysis were included in the multivariate logistic regression analysis. Multivariate analysis using stepwise forward selection was used to create a logistic proportional hazards model to determine the independent risk factors for ADV coinfection or drug-resistant MP infection. The inclusion criterion for the factors was a *P* < 0.05. The sensitivity and specificity of the model were evaluated using receiver operating characteristic (ROC) curve analysis. Analyses were performed using SPSS v22.0.

To determine the optimal cut-off value for age for assessing the risk of viral coinfection, we applied a classification tree. This machine learning tree-based approach applied binary recursive partitioning for age. The recursion was completed when splitting no longer added value to the prediction of the risk of coinfection.

## Results

### Clinical characteristics of patients

**Fig. 1:**
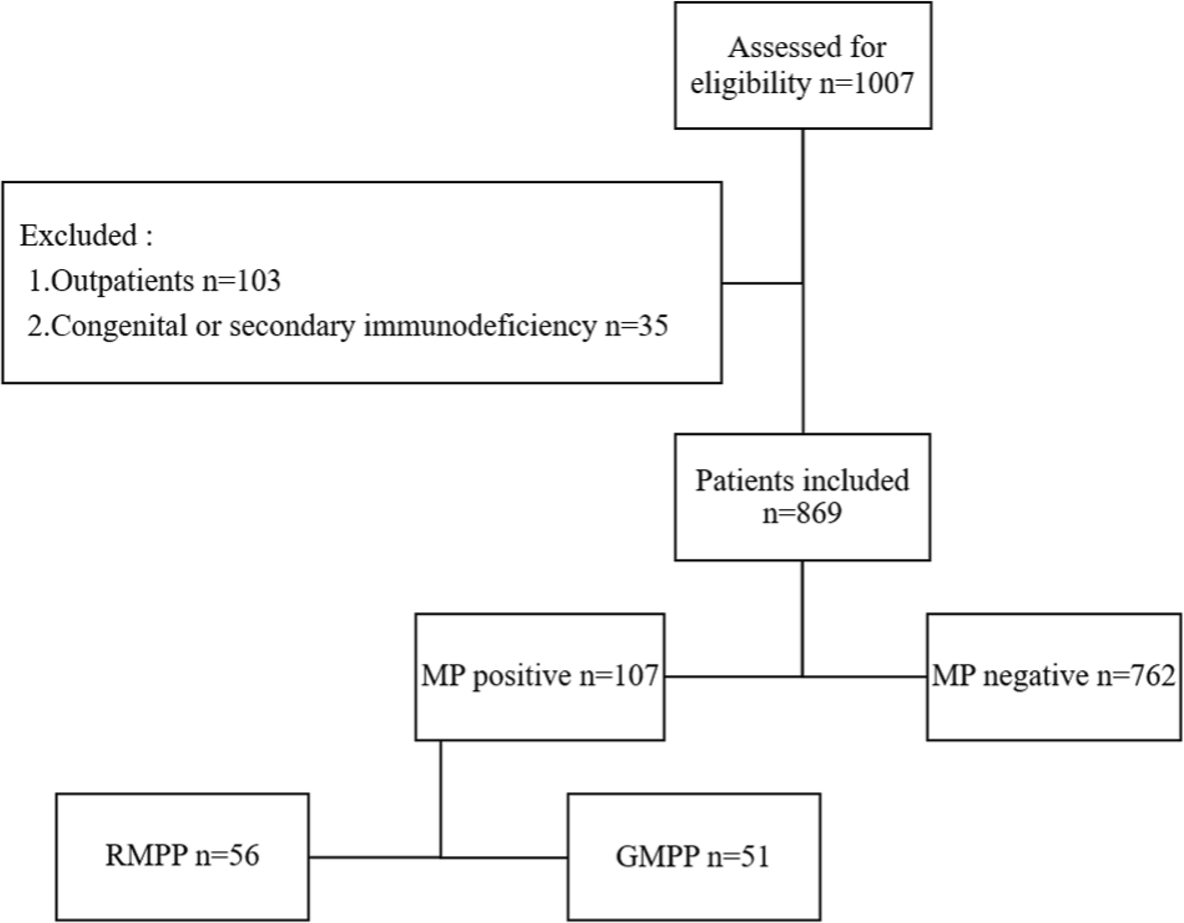
Flow diagram illustrating the design of the present study

A total of 107(10.63%,107/1007) patients diagnosed with MPP, aged 81 days to 14 years, were enrolled in our study between December 1, 2016, and May 31, 2019. Of these, 55 patients were male. MP-IgM of 83 patients were positive during the study period. The study design is illustrated in Fig. 1. There was no significant difference in gender. Fifty-six patients were diagnosed with RMPP. The general characteristics of all 107 patients are shown in Table 1.

**Table 1:** General characteristics of the patients

Characteristic	No (%)
Total	107
Age (y)	4.268 ± 3.294
Gender, No. (%)	
Male	55(51.40)
Female	52(48.60)
Underlying diseases, No. (%)	
None	98(91.59)
Congenital heart disease	5(4.67)
Congenital biliary atresia	2(1.87)
Other diseases	2(1.87)
Symptoms and signs	
Fever, No. (%)	100(93.46)
Duration of fever (d)	7.690 ± 5.770
Cough, No. (%)	107(100)
Wheezing, No. (%)	20(18.69)
Duration of wheezing (d)	0.00(0.00,5.00)
RMPP, No. (%)	56(52.34)
Hypoxemia, No. (%)	16(14.95)
Mechanical ventilation, No. (%)	7(6.54)
Application of glucocorticoids, No. (%)	60(56.07)
Replacement of antibiotics, No. (%)	19(17.76)
7-day course of azithromycin, No. (%)	42(39.25)
Coinfection, No. (%)	60(56.07)
MP with A2063G mutation, No. (%)	60(56.07)
Prognosis after treatment, No. (%)	
Surviving	105(98.13)
Nonsurviving	2(1.87)

Data are No. (%) of patients, mean ± standard deviation (SD) or median (interquartile range)

### Distribution and prevalence of pathogens

**Fig. 2:**
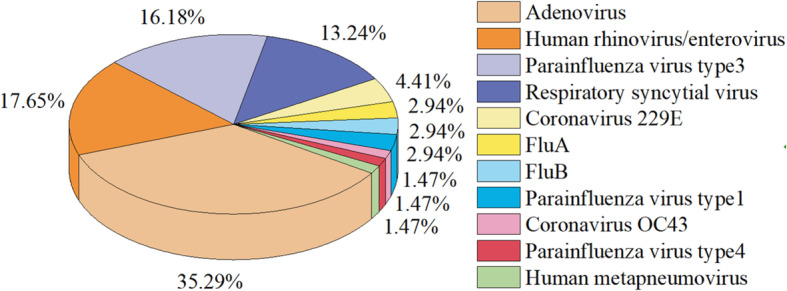
Pathogens detected by FilmArray RP

Among the 107 patients, 60 patients were coinfected with viruses, including human rhinovirus/enterovirus, ADV, RSV, parainfluenza virus type 1, parainfluenza virus type 3, parainfluenza virus type 4, coronavirus OC43, coronavirus 229E, FluA and FluB. The overall coinfection rate was 56.07%. ADV was the most prevalent organism, accounting for 22.43% (24/107), followed by parainfluenza virus (13.08%,14/107) and human rhinovirus/enterovirus (11.21%, 12/107) (Fig. 2). Two or more pathogens were detected in 11 patients, and the rate of infection with multiple viruses was 10.28%. Coinfections were more likely to be found in younger patients. Three years old may be an optimal cut-off value to distinguish viral coinfection from MP single infection using the machine learning-based method, the classification tree.

All patients enrolled in this study underwent A2063G and A2064G mutation gene tests with PCR. Sixty (60/107, 56.07%) patients were confirmed to have a gene mutation, all of which were A2063G mutations, the most prevalent mutation in Asian countries. Among the 60 patients infected with drug-resistant MP, 40 (40/60, 66.67%) progressed to RMPP. However, only 16 (16/47, 34.04%) children infected by drug-sensitive MP were diagnosed with RMPP.

### Clinical characteristics of general MPP and RMPP

Fifty-six (56/107, 52.34%) patients were diagnosed with RMPP during our research period. A2063G mutation and coinfection with ADV were more commonly detected in the RMPP group (*P* = 0.001; *P* = 0.019). More patients with RMPP continued having a fever after receiving macrolide antibiotic treatment for 48 h (*P* < 0.001), leading to a significantly longer duration of fever (*P* < 0.001) and increasing the proportion of patients using glucocorticoids (*P* = 0.001). Although there were no significant differences in the leukocyte counts and CRP levels, the erythrocyte sedimentation rate (ESR) was higher in the RMPP group (*P* = 0.045). There were no remarkable differences in the total coinfection rate and single virus coinfection rate between the two groups, except for the patients with ADV coinfection (*P* = 0.019). ADV coinfection was more likely to be detected in patients with RMPP. With respect to the lung radiographs, more pulmonary consolidation was found in the patients with RMPP. In contrast, interstitial changes were likely to be discovered in general MPP (GMPP) (*P* = 0.02) (Table 2).

**Table 2:** Clinical characteristics of GMPP and RMPP

Characteristics	GMPP (*n* = 51)	RMPP (*n* = 56)	*P* value
Age (y)	3.679 ± 3.193	4.805 ± 3.322	0.077
Gender (male/female)	27/51	28/56	0.847
Clinical presentation			
Wheezing, No. (%)	7(13.73)	13(23.21)	0.227
Hypoxemia, No. (%)	4(7.84)	12(21.43)	0.060
Duration of fever (d)	4.784 ± 2.831	10.339 ± 6.473	< 0.001
Duration of wheezing (d)	0.00(0.00,5.00)	0.00(0.00,7.00)	0.459
Extrapulmonary complications, No. (%)	6(11.76)	12(23.21)	0.137
Imaging features, No. (%)			
Consolidation, No. (%)	22(43.14)	37(66.07)	0.02
Interstitial changes, No. (%)	26(50.98)	16(28.57)	0.029
Laboratory detection			
White blood cell count (*10^9^/L)	9.583 ± 3.348	8.420 ± 3.530	0.084
Lymphocyte percentage (%)	37.291 ± 14.817	32.352 ± 14.690	0.088
C-reactive protein (mg/l)	12.00(8.00,25.00)	11.00(5.00,25.75)	0.664
ESR (mm/h)	29.11 ± 19.935	37.88 ± 22.412	0.045
Coinfection, No. (%)	28(54.90)	32(57.14)	0.847
Coinfection with adenovirus, No. (%)	6(11.76)	18(32.14)	0.019
A2063G mutation, No. (%)	20(39.22)	40(71.43)	0.001
Treatment			
No fever within 48 h after application of macrolides, No. (%)	6(11.76)	50(89.29)	< 0.001
Application of glucocorticoids, No. (%)	24(47.06)	44(78.57)	0.001
Mechanical ventilation, No. (%)	4(7.84)	3(5.36)	0.707

Data are No. (%) of patients, mean ± standard deviation (SD) or median (interquartile range)

Abbreviation: General *Mycoplasma pneumoniae* pneumonia (GMPP); refractory *Mycoplasma pneumoniae* pneumonia (RMPP)

### Predictors of adenovirus coinfection or drug-resistant MP infection

As mentioned above, more children coinfected with ADV or infected with drug-resistant MP were found in the RMPP group than in the GMPP group. It is important to investigate the risk factors for ADV coinfection or macrolide-resistant MP infection. During the study period, 24 (24/107, 22.43%) patients were coinfected with ADV, which made the course of the disease more severe. Nine (9/24, 37.5%) patients who developed extrapulmonary complications were coinfected with ADV, whereas only 10 (10/83, 12.05%) children were diagnosed with extrapulmonary complications in the non-ADV group (*P* = 0.012). Wheezing and lung consolidation were more likely to occur in children with ADV coinfection than in those without (*P* = 0.002; *P* = 0.010). Furthermore, the duration of fever in ADV-coinfected patients was much longer than in the other patients (*P* = 0.007) (Table 3).

**Table 3:** Risk factors for MPP with adenovirus coinfection

Characteristics	With adenovirus coinfection (*n* = 24)	Without adenovirus coinfection (*n* = 83)	*P* value
Age (y)	3.362 ± 2.590	4.531 ± 3.440	0.078
Gender (male/female)	13/11	42/41	0.819
Clinical presentation			
Wheezing, No. (%)	10(41.67)	10(12.05)	0.002
Hypoxemia, No. (%)	6(25.00)	10(12.05)	0.189
Extrapulmonary complications, No. (%)	9(37.50)	10(12.05)	0.012
Duration of fever (d)	12.083 ± 9.343	6.422 ± 3.357	0.007
Duration of wheezing (d)	0.00(0.00,8.00)	0.00(0.00,5.00)	0.570
Fever longer than 7 days, No. (%)	20(83.33)	36(43.37)	0.001
Imaging features			
Consolidation, No. (%)	19(79.17)	40(48.19)	0.010
Interstitial changes, No. (%)	5(20.83)	37(44.58)	0.056
Laboratory tests			
White blood cell count (*10^9^/L)	9.003 ± 3.487	8.966 ± 3.496	0.964
Lymphocyte percentage (%)	36.488 ± 14.274	34.153 ± 15.106	0.491
C-reactive protein (mg/l)	10.00(4.00,25.00)	12.00(7.25,25.00)	0.997
A2063G mutation, No. (%)	9(37.50)	51(61.45)	0.060
Treatment			
No fever within 48 h after application of macrolides, No. (%)	5(20.83)	26(31.33)	0.445
Application of glucocorticoids, No. (%)	17(70.83)	51(61.45)	0.476
Mechanical ventilation, No. (%)	1(4.17)	6(7.23)	1

Data are No. (%) of patients, mean ± standard deviation (SD) or median (interquartile range)

To identify drug-resistant MP infection, mutant gene detection was carried out. The total positive rate was 56.07%. The mutation rate was 71.43% in RMPP patients, which was significantly higher than that in the GMPP group (*P* = 0.001) (Table 2). More patients with prolonged fever duration after the appropriate administration of macrolides for 48 h were in the group infected by drug-resistant MP than in the group not infected with drug-resistant MP (*P* = 0.002) (Table 4). In addition, a fever duration longer than 7 days had a strong relationship with drug-resistant MP infection according to multivariable logistic regression (OR = 3.500, 95% CI = 1.310–9.353, *P* = 0.012).

**Table 4:** Risk factors for MPP with A2063G mutation

Characteristics	Without A2063G mutation (*n* = 47)	With A2063G mutation (*n* = 60)	*P* value
Age (y)	3.792 ± 3.537	4.642 ± 3.069	0.187
Gender (male/female)	23(58.97)	32(53.33)	0.699
Clinical presentation			
Wheezing, No. (%)	10(25.64)	10(16.67)	0.621
Hypoxemia, No. (%)	8(20.51)	8(13.33)	0.599
Extrapulmonary complications, No. (%)	9(23.08)	10(16.67)	0.802
Duration of fever (d)	7.234 ± 6.356	8.050 ± 5.293	0.470
Duration of wheezing (d)	0.00(0.00,6.50)	0.00(0.00,5.00)	0.499
Fever longer than 7 days, No. (%)	16(41.03)	35(56.33)	0.019
Imaging features, No. (%)			
Consolidation, No. (%)	30(76.92)	29(48.33)	0.122
Interstitial changes, No. (%)	16(41.03)	26(43.33)	0.425
Laboratory tests, No. (%)			
White blood cell count (*10^9^/L)	9.274 ± 3.179	8.740 ± 3.701	0.433
Lymphocyte percentage (%)	35.023 ± 15.922	34.410 ± 14.142	0.834
C-reactive protein (mg/l)	13.00(8.00,29.00)	11.00(4.50,22.00)	0.107
ESR (mm/h)	29.950 ± 21.995	36.620 ± 21.102	0.136
Treatment			
No fever within 48 h after application of macrolides, No. (%)	21(53.85)	10(16.67)	0.002
Application of glucocorticoids, No. (%)	24(61.54)	44(73.33)	0.026
Mechanical ventilation, No. (%)	4(10.26)	3(5.00)	0.697

Data are No. (%) of patients, mean ± standard deviation (SD) or median (interquartile range)

**Fig. 3:**
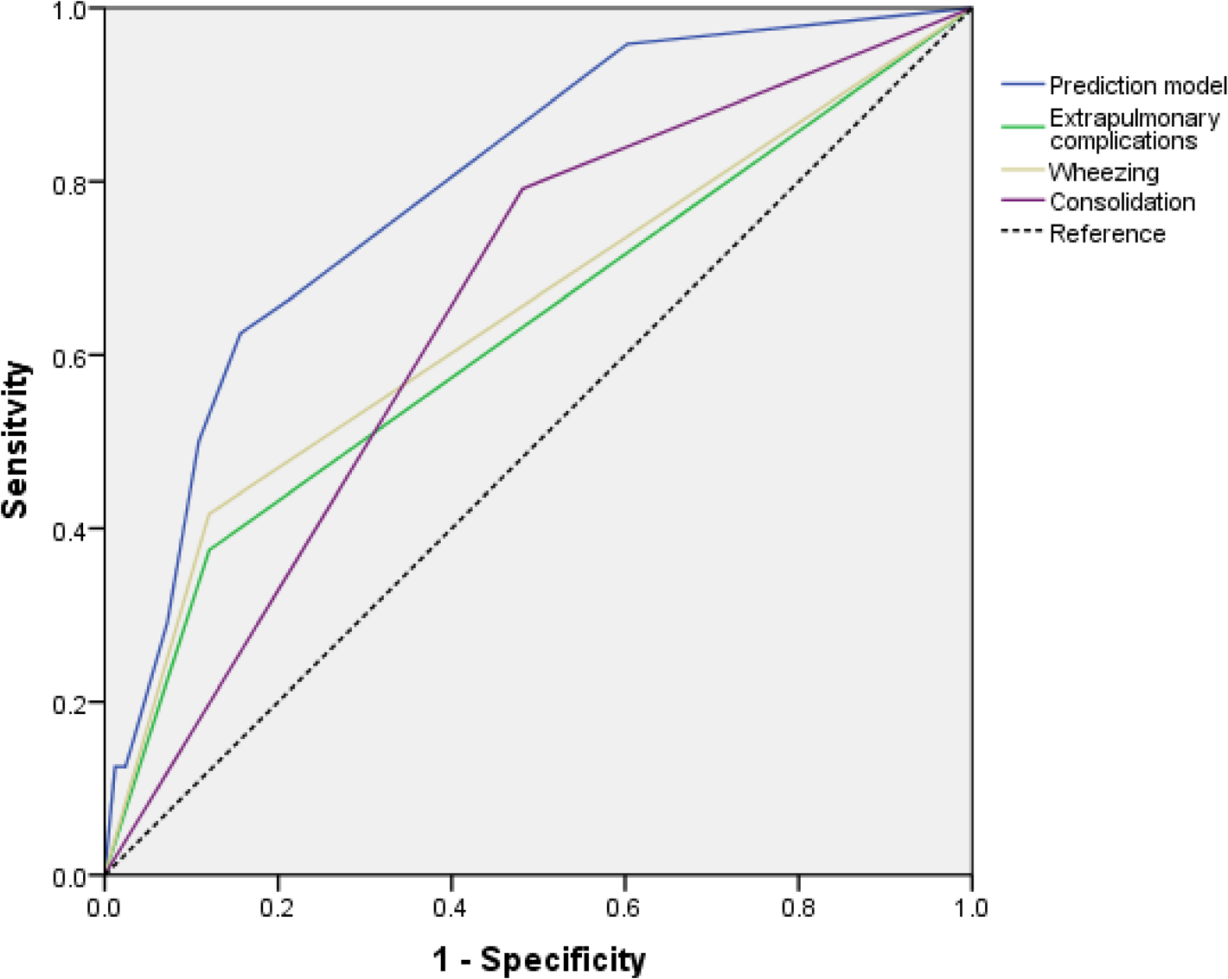
Receiver operating characteristic (ROC) analysis showing the power of the risk factors and prediction model for the prediction of adenovirus coinfection. The area under the ROC curve (AUC) of the prediction model for the prediction of adenovirus coinfection was 0.795

Multivariable logistic regression was further carried out to verify the associations between the variables and ADV coinfection. Patients with symptoms of wheezing (X_1_) (OR = 5.559, 95% CI = 1.726–17.901, *P* = 0.004), consolidation on chest X-ray (X_2_) (OR = 4.290, 95% CI = 1.305–14.106, *P* = 0.016) and extrapulmonary complications (X_3_) (OR = 4.225, 95% CI = 1.331–13.603, *P* = 0.015) all had a higher risk of ADV coinfection than their counterparts (Table 5). Therefore, based on logistic regression, a prediction model including wheezing, lung consolidation and extrapulmonary complications was established: Logit(P) = (− 2.931) + 1.715X_1_ + 1.456X_2_ + 1.488X_3_. The predictive values of the individual risk factors and the prediction model were assessed by ROC curve analysis, which showed that the model had a high level of diagnostic accuracy, with an AUC of 0.795 (95% CI 0.679–0.893, *P* < 0.001). Additionally, wheezing, lung consolidation and extrapulmonary complications had some predictive value separately (Fig. 3).

**Table 5:** Analysis of risk factors related to adenovirus coinfection

Variables	OR (95%)	*P* value
Wheezing	5.559(1.726–17.901)	0.004
Lung consolidation	4.290(1.305–14.106)	0.016
Extrapulmonary complications	4.225(1.331–13.603)	0.015

## Discussion

MP is a common pathogen that causes community-acquired pneumonia in children [1]. The proportion of pneumonia caused by MP in different studies ranged from 20 to 40% [14]. In the present study, the MP infection rate was 10.63%. Besides, the MP infection rate may increase with age according to Li’s research [12]. MP-IgM may not be detectable in the very early stage of the disease, which may explain why only 83 patients were MP-IgM positive in the present study. This result suggests that combining MP-IgM and RT-PCR could increase the diagnostic accuracy of mycoplasma infection in children, similar to the findings of the study by Biljana Medjo’s [15]. In general, viral coinfection rates in children with MPP ranged from 10 to 30% [16–20]. In the present study, 56.07% of patients were coinfected with at least one type of virus, which was higher than the proportions reported in other studies, possibly because of different climates and races. Additionally, the sensitivity and accuracy of the FilmArray respiratory pathogen panel contributed to a higher positive rate as well. Viral coinfections were more likely to be found in relatively younger children, especially those under 3 years old, which is similar to the results of Zhang’s research [7].

It should be noted that ADV coinfection and drug-resistant MP infection were more common in the RMPP group than in the GMPP group. A previous study showed that compared with RMPP children without coinfections, those who were coinfected with viruses and bacteria had more severe disease [20]. Thus, respiratory viral infection may lead to the development of RMPP, and coinfection might result in further progression of the disease. However, in this study, no remarkable differences in clinical characteristics were observed between patients who were and were not coinfected, except for in those with ADV coinfections.

ADV infection may cause severe disease necessitating ICU admission and mechanical ventilation [21]. The severity of pneumonia with ADV coinfection is significantly related to viral load and serotypes, and children with ADV genotype 7 develop severe pneumonia more frequently than those with other genotypes [22]. However, the effect of ADV coinfection on MPP in children remains unclear. According to the present results, this is the first study to report that ADV coinfection led to more severe disease severity in children with MPP and increased the proportion of children with RMPP. Furthermore, a prediction model including wheezing, lung consolidation and extrapulmonary complications was established to predict ADV coinfection in children with MPP. This prediction model can help clinicians identify a severe disease course of MPP early, which is beneficial for the precise selection of medications.

Drug resistance is another inevitable factor contributing to the development of RMPP [8]. Mutations at position 2063 or 2064 domain V in the 23S rRNA gene are considered to be related to macrolide resistance [22, 23]. The mutation rate in this study was 56.07%, of which were all A2063G mutations. The rate of drug-resistant MP was lower than those reported in other studies in China, where the rates range from almost 70 to 90% [21, 22, 24]. This is the because point mutations in drug-resistance genes were detected by PCR in this study, while drug sensitivity tests were chosen by other researchers. In addition, racial differences also play an important role in drug-resistant MP infection. Macrolide resistance is less common in the United States and European countries, where the macrolide-resistant MP prevalence is below 30% [23, 25, 26]. A relatively high mutation rate in China is probably related to immoderate exposure to macrolides, since they are widely used for the treatment of respiratory infections in outpatients, especially in children. Therefore, alternative medicines are urgently needed.

A study from Japan reported that the macrolide-resistance rate decreased by 59.3% in 2014 and 43.6% in 2015 from the highest macrolide-resistance rate of 81.6% in 2012 [27]. Guidelines published by the Japan Society of Pediatric Pulmonology/Japanese Society for Pediatric Infectious Diseases recommend tosufloxacin, which was approved for pediatric use in Japan in 2010, as a second-line drug when patients have a fever for 2–3 days after the administration of macrolides [28]. Hence, the decrease in the drug-resistance rate during this period may be attributed to the decrease in the use of oral macrolides. In children with drug-resistant MPP, tetracyclines (doxycycline, minocycline) have shown excellent efficacy [29–31]. Tetracyclines are well known for causing adverse reactions, including gastrointestinal disturbances, esophagitis, photosensitivity, and tooth discoloration [32]. Because of these adverse reactions, tetracyclines are contraindicated in pregnant women and children under 8 years old. However, previous studies showed that short cycles and limited courses of treatment (fewer than 6 courses, 6 days per course) caused insignificant tooth discoloration in children under 5 years old [33]. This study showed that drug-resistant MP infection contributes to the development of RMPP. On the other hand, high MP-DNA copy numbers might indicate a severe disease course in MPP children. A previous study showed that a reduced MP-DNA copy number in the sputum was well correlated with patients’ clinical symptoms and the therapeutic efficacy of antibiotics [34]. Studies have shown that levofloxacin and minocycline therapy in patients who are nonresponsive to macrolides may reduce their fever duration and result in a more rapid decrease in the sputum MP-DNA copy number [35, 36]. Despite the efficacy of fluoroquinolones, clinicians should be aware of the adverse reactions (musculoskeletal adverse events), and the usage of fluoroquinolones should be based on in vitro activity and disease severity in children with MPP.

As mentioned above, it was found that patients infected with drug-resistant MP have a longer fever duration, and more of them maintained a fever for more than 2 days after the appropriate application of macrolides. Hence, switching antibiotics should be considered when a patient still has a fever after the administration of macrolides for 2 days. A long fever duration of 7 days may strongly suggest infection with drug-resistant MP, which indicates that changing to a more effective antibiotic is urgently required. In our department, the policy for changing antibiotics is as follows: 1. if the patient is infected with MP only or coinfected with virus, based on laboratory tests and clinical symptoms, antibiotics are not changed until the first 5-day course is finished, with the prerequisite that the patient is not seriously ill; 2. if the patient has coinfection with other bacteria, we usually combine penicillin or cephalosporin with a macrolide. For patients with drug-resistant MP infections or whose condition worsens (sustained fever, deterioration on lung imaging or worsening clinical symptoms), minocycline is the first consideration for patients older than 8 years, while for those younger than 8 years, levofloxacin is applied only in case of severe pneumonia.

Nevertheless, this study has several limitations. First, this study was performed at a single center, and the distribution of pathogens was greatly influenced by the region and climate. In addition, the sample size was relatively small. Furthermore, the immune conditions of the patients were not investigated in our research, and immunity may affect the severity of MPP.

## Conclusions

The occurrence of RMPP is associated with ADV coinfection and drug-resistant MP infection. A prediction model combining wheezing, extrapulmonary complications and lung consolidation can be used to predict ADV coinfection in children with MPP. Viral coinfections are more common in MPP patients under 3 years old. Macrolide-resistant MP infection should be taken into consideration when a patient continues to have a fever after the administration of appropriate macrolide antibiotics for 2 days. A 7-day fever duration despite the use of macrolide antibiotics strongly suggests that the patient is infected by drug-resistant MP, and a timely switch to another effective antibiotic should be considered after the potential adverse reactions to the medicine and severity of disease are evaluated.

## Data Availability

The datasets used and/or analyzed during the current study are available from the corresponding author on reasonable request.

## Abbreviations

ADV: Adenovirus
*B. pertussis*: *Bordetella pertussis*
*C. pneumoniae*: *Chlamydophila pneumoniae*
CAP: Community-acquired pneumonia
CoV: Coronaviruses
FilmArray RP: FilmArray Respiratory Panel
FluA: Influenza A viruses
FluB: Influenza B viruses
hMPV: Human metapneumovirus
*M. pneumoniae*: *Mycoplasma pneumoniae*
MP: *Mycoplasma pneumoniae*
MPP: *Mycoplasma pneumoniae* pneumonia
RMPP: Refractory *mycoplasma pneumoniae* pneumonia
NPS: Nasopharyngeal swab
Para: Parainfluenza virus
PCR: Polymerase chain reaction
Rhino/ Entero: Human rhinovirus/enterovirus
RSV: Respiratory syncytial virus
SCMC: Shanghai Children’s Medical Center
